# Naloxone Co-prescription in Chronic Opioid Therapy: Benefits, Pitfalls, and Lessons Learned

**DOI:** 10.7759/cureus.94570

**Published:** 2025-10-14

**Authors:** Alexander Kim, Scott Weiner

**Affiliations:** 1 Anesthesiology, Brigham and Women's Hospital, Harvard Medical School, Boston, USA; 2 Emergency Medicine, Brigham and Women's Hospital, Harvard Medical School, Boston, USA

**Keywords:** chronic opioids, naloxone education, opioid management, prescription opioid overdose deaths, stigma in health care

## Abstract

There has been a well-publicized epidemic of lethal and non-lethal prescription opioid overdose in the United States. Significant efforts have been made to alter this trajectory, both at the public health and direct medical care levels. One specific method of harm reduction has been the co-prescription of intranasal naloxone to all patients who are prescribed chronic opioids. In this editorial, we discuss our experience of instituting this quality improvement initiative within a large academic pain management center. After an adverse event of inadvertent administration of intranasal naloxone precipitating opioid withdrawal and emergency evaluation, we learned several lessons. These lessons include cultural sensitivity, being specific on prescription instructions, and how to best discuss naloxone co-prescription with our patients.

## Editorial

In the wake of the well-publicized epidemic of lethal and non-lethal prescription opioid overdose in the United States, significant efforts have been made to alter this trajectory, both at the public health and direct medical care levels. Despite efforts to identify high-risk patients on chronic opioid therapy, such as utilizing urine toxicology screens or screening tools, e.g., Current Opioid Misuse Measure (COMM), it remains difficult to predict which individual patients will suffer an overdose [1]. It is known that overdose risk exponentially increases as certain thresholds of daily morphine milligram equivalents (MME) are surpassed [2]. One method of harm reduction is the co-prescription of intranasal naloxone in a Universal Precautions-type model [3]. Naloxone is a high-affinity, competitive opioid antagonist, particularly at the mu receptor, which is responsible for the respiratory depressant effects of opioid medications. In cases of suspected opioid overdose, administration of intranasal naloxone may displace opioid agonists at the receptor, thus reversing symptoms of overdose generally within 10 minutes [4]. It has been demonstrated that the co-prescription of naloxone along with chronic opioid therapy may reduce the risk of opioid-related adverse effects, such as opioid-related emergency department (ED) visits [5].

At our large academic medical center, the multidisciplinary pain management team implemented a quality improvement initiative to co-prescribe intranasal naloxone for all patients on chronic opioid therapy at or exceeding 60 daily MME. This MME threshold was associated with increased risk of opioid overdose-related mortality, while noted to be commonly within clinical practice [2]. Once these individuals were identified, which included over 600 distinct patients, a letter was sent to them describing the quality improvement initiative, the potential risks of opioid medications, and the potentially life-saving role of having naloxone available. To emphasize that no patients were being singled out and to avoid assigning unintentional stigma of opioid misuse, the letter clearly explained that a naloxone prescription would be written for all patients at or surpassing the predefined daily MME.

Several months after this initiative was undertaken, our pain management center was notified that a patient had suffered harm in relation to the intranasal naloxone prescription. By report, the patient had filled the prescription and, upon receiving the medication, had applied it intranasally and had symptoms of precipitated opioid withdrawal. Her family had called emergency medical services, and the patient had been restrained due to combative behavior and suffered abrasions and soft tissue contusions. The family also reported that once in the ED, she had felt stigmatized and thought of as a patient with a substance use history, before eventually being discharged. After debriefing with the patient’s daughter the next day, it was determined that a number of factors contributed to this incident. The patient's primary language was Spanish; she had mild cognitive impairment and believed that the intranasal naloxone was fluticasone nasal spray. She also reported not having been aware of the letter regarding the quality improvement initiative. Furthermore, the letter was written in English. Another issue, which was immediately dealt with after the event, was that the default instruction for the naloxone prescription read “1 spray by intranasal route once for 1 dose”. It was subsequently changed to an as-needed basis, and a more detailed instruction was given: “1 spray by intranasal route in case of suspected overdose”.

Based on this experience, we suggest that the intranasal naloxone co-prescription be written by the longitudinal opioid prescriber, accompanied by a thorough conversation detailing the rationale. This messaging would likely have been better received and understood coming from a physician with whom a therapeutic relationship has been fostered, rather than an impersonal letter. Indeed, other patients felt displeased by this prescription being written by someone who was not “their doctor” and reported feeling stigmatized, despite our efforts to do the opposite. Language barriers and healthcare literacy must be addressed as well. As it is a medical treatment, the ethical concepts of informed consent and shared decision-making need to be exercised. Ultimately, we are optimistic that we have improved the safety of this cohort of patients and would be pleased if even a single overdose event were prevented or effectively treated. However, we share this experience to help avoid adverse outcomes, such as unintentional precipitated opioid withdrawal, for other practices considering this important intervention.

## References

[1] Ferris LM, Saloner B, Krawczyk N (2019). Predicting opioid overdose deaths using prescription drug monitoring program data. Am J Prev Med.

[2] Gomes T, Mamdani MM, Dhalla IA, Paterson JM, Juurlink DN (2011). Opioid dose and drug-related mortality in patients with nonmalignant pain. Arch Intern Med.

[3] Takeda MY, Katzman JG, Dole E, Bennett MH, Alchbli A, Duhigg D, Yonas H (2016). Co-prescription of naloxone as a Universal Precautions model for patients on chronic opioid therapy - observational study. Subst Abus.

[4] Chou R, Korthuis PT, McCarty D (2017). Management of suspected opioid overdose with naloxone in out-of-hospital settings: a systematic review. Ann Intern Med.

[5] Coffin PO, Behar E, Rowe C, Santos GM, Coffa D, Bald M, Vittinghoff E (2016). Nonrandomized intervention study of naloxone coprescription for primary care patients receiving long-term opioid therapy for pain. Ann Intern Med.

